# Bilosomes and Niosomes for Enhanced Intestinal Absorption and In Vivo Efficacy of Cytarabine in Treatment of Acute Myeloid Leukemia

**DOI:** 10.3390/ph17121572

**Published:** 2024-11-23

**Authors:** Abdelrahman R. Said, Mona F. Arafa, Walaa A. El-Dakroury, Sultan Alshehri, Gamal M. El Maghraby

**Affiliations:** 1 Department of Pharmaceutical Technology, Faculty of Pharmacy, Tanta University, Tanta 31527, Egypt; mona.arafa@pharm.tanta.edu.eg (M.F.A.); gamal.elmaghraby@pharm.tanta.edu.eg (G.M.E.M.); 2 Department of Pharmaceutics and Industrial Pharmacy, Faculty of Pharmacy, Badr University in Cairo (BUC), Badr City 11829, Egypt; 3 Department of Pharmaceutics, Faculty of Pharmacy, University of Tabuk, Tabuk 71491, Saudi Arabia; 4 Department of Pharmaceutics, College of Pharmacy, King Saud University, Riyadh 11451, Saudi Arabia; salshehri1@ksu.edu.sa; 5 Faculty of Pharmacy, Alsalam University, Tanta 31527, Egypt

**Keywords:** bilosomes, niosomes, intestinal perfusion, leukemia, cytarabine

## Abstract

Cytarabine (CTR) is a hydrophilic anticancer drug used to treat leukemia. It suffers from poor permeability and intestinal metabolism, diminishing its oral bioavailability. Background/Objectives: The objective was to develop and evaluate niosomes and bilosomes for enhanced intestinal absorption; hence, oral bioavailability. Results: CTR-loaded niosomes and bilosomes with vesicle sizes of 152 and 204.3 nm were successfully prepared with acceptable properties. The presence of bile salts increased the zeta potential of bilosomes. The recorded entrapment efficiency of cytarabine was acceptable for such a hydrophilic drug. CTR-bilosomes showed a pH-dependent drug release pattern with preferred release in pH 6.8. Intestinal absorption behavior indicated a site-dependent CTR absorption pattern with unfavorable absorption in the distal intestine. Niosomal and bilosomal formulations enhanced intestinal absorption parameters with evidence for a predominant paracellular absorption mechanism that bypasses intestinal barriers. The investigation of the anti-leukemic effect of niosomal and bilosomal formulations indicated that both formulations ameliorated the blood parameters, reflecting significant improvement in leukemia treatment compared with the drug solution. Pathological examination of blood films revealed decreased blast cells in peripheral blood in groups treated with tested formulations. Methods: Tested formulations were prepared according to the pro-concentrate method and characterized for particle size, zeta potential, entrapment efficiency, and in vitro release. CTR-loaded niosomes and bilosomes were evaluated for enhanced intestinal absorption utilizing the single-pass in situ intestinal perfusion method in rabbits, and the anti-leukemic effect was assessed using the benzene-induced leukemia model in rats. Conclusions: This study introduced surfactant vesicles for enhanced oral bioavailability of CTR.

## 1. Introduction

Cytarabine (CTR) is an FDA-approved antineoplastic agent commonly used in acute and chronic leukemia treatment regimens, particularly effective against acute myeloid leukemia (AML) and acute lymphocytic leukemia (ALL) when combined with anthracyclines [1]. CTR is an anti-metabolic agent converted intracellularly to cytarabine triphosphate, which kills abnormal cells by damaging DNA. Its structural similarity to deoxycytosine enables its incorporation into the cell cycle, interfering with DNA synthesis and inducing cell death [2]. Also, it can inhibit the activity of DNA polymerase, which has a significant role in cell division and proliferation [3]. Unfortunately, this drug is bioavailable only after intravenous and subcutaneous administration [4]. Oral use of CTR is restricted due to the extensive first-pass metabolism by hepatic and intestinal cytochrome P-450 enzymes inside intestinal cells as well as efflux pump via P-glycoproteins inside intestinal cells [5]. The fast deamination of CTR into an inactive form by intracellular cytidine deaminase, along with other factors, is the primary reason for CTR’s poor oral bioavailability. Moreover, CTR is a highly hydrophilic molecule contributing to reduced oral bioavailability due to limited membrane permeability [6].

Several attempts have been made to overcome these problems by chemical modification or incorporation into various pharmaceutical formulations to enhance CTR oral bioavailability. Many investigators presented remarkable efforts to modify CTR to other prodrugs with better biopharmaceutical properties chemically. This involved conjugation with amino acids (e.g., valine and phenylalanine) or fatty acids (elaidic acid, myristic acid, or palmitic acid) besides the phosphate derivatives, which were extensively investigated because of their ability to bypass the intracellular phosphorylation steps [7,8,9,10]. Another approach for improving the bioavailability of CTR applied recent drug delivery systems. Nano-formulations such as gelatin nanocarriers, nanoparticulate systems, poly (propylene imine) dendrimers, sodium N-lauryl amino acid cationic aggregates, and, most importantly, vesicular systems such as liposomes and niosomes were introduced as promising candidates for the enhancement of the oral bioavailability of CTR [11].

Liposomes encapsulating CTR offered better pharmacokinetic properties of this drug, as intact vesicles were absorbed through the intestinal membrane to the reticuloendothelial system, minimizing intestinal and hepatic deamination, besides the fact that intact vesicles are retained more effectively in tumor tissue [12]. The most valuable alternative to liposomes is niosomes, which are composed of non-ionic surfactants with cholesterol as the principal component.

Niosomes are vesicles composed of non-ionic surfactants that function as carriers for drug delivery. Structurally, these entities resemble liposomes; however, they are composed of non-ionic surfactants instead of phospholipids, resulting in enhanced stability and cost-effectiveness. Niosomes are characterized by an aqueous core encased in one or more layers of amphiphilic molecules, enabling the encapsulation of hydrophilic and hydrophobic drugs [13]. Their distinct composition offers enhanced drug stability, controlled release, and improved bioavailability, rendering them appropriate for targeted and sustained drug delivery applications [14]. Niosomes share all favorable properties of liposomes, including the in vivo properties of modified drug disposition, while offering better stability and low cost [13]. CTR-loaded niosomes have been previously developed; however, the authors focused on monitoring entrapment efficiency, release, and storage effects only, leaving the delivery potential via the oral route unexplored; niosomes loaded with cytarabine hydrochloride exhibited sustained release, achieving approximately 80% drug entrapment efficiency [15]. This slow-release profile is expected to maintain more stable blood concentrations of cytarabine if explored in vivo, which may improve therapeutic efficacy and reduce adverse effects. Furthermore, niosomal encapsulation enhances medication stability in gastrointestinal environments and the oral bioavailability of numerous medicines and maintains therapeutic levels for extended durations relative to free medication [16]. Due to their lipid composition, niosomes can improve the bioavailability of orally administered medications through a process akin to that proposed for conventional lipid-based carriers. The fluidization of the membrane, followed by the enhancement of the transcellular route, may be regarded as the initial potential approach. Alternative strategies may rely on the influence of lipid digestion products that enhance the paracellular route by expanding the tight junctions. The final theory employs the potential translymphatic absorption of intact niosomes. The subsequent mechanism offers an added benefit by circumventing the presystemic disposition [17].

Bilosomes are modified vesicular systems that contain bile salts inside the bilayer structure of the vesicles. Incorporating bile salts as a bilayer component acts as a fluidizing agent, engages with lipid bilayers, integrates into the membrane, and augments fluidity, diminishing membrane stiffness, becoming more hydrated, and improving permeability for specific solutes [18,19]. Bilosomes demonstrate the potential for enhancing the oral bioavailability of poorly absorbed drugs by utilizing their capacity to withstand digestive enzyme degradation and enhance membrane permeability. Research has shown that bilosomal carriers enhance bioavailability and facilitate sustained release for various pharmaceuticals. Cytarabine conjugated with bile acids demonstrates a two-fold increase in bioavailability, attributed to enhanced absorption through transporter targeting. [20]. Furthermore, similar drugs utilizing bilosome systems demonstrated improved bioavailability, attributed to enhanced permeability and stability under gastrointestinal conditions [21]. The most common techniques for producing bilosomes are thin-film hydration, ethanol injection, reverse-phase evaporation, and the pro-concentrate method [22]. The pro-concentrate method is considered the most suitable technique as it improves the stability of bilosomes, particularly during storage, and has proven to enhance the oral bioavailability of medications such as cyclosporine A compared to conventional bilosome formulations, demonstrating superior absorption rates in pharmacokinetic studies [23].

Niosomes and bilosomes provide a promising approach to enhance cytarabine’s bioavailability by facilitating improved oral absorption, potentially making it more effective as an oral therapeutic agent.

Accordingly, this work aimed to investigate the impact of CTR encapsulation into niosomes and bilosomes on intestinal absorption. This employed an in situ rabbit single-pass intestinal perfusion model. This model can identify the intestinal absorption characteristics and determine the primary site of intestinal absorption of CTR [24]. This study was extended to evaluate the effect of CTR loading into niosomes and bilosomes on the in vivo efficacy of the drug. The latter employed a leukemia model induced in rats. This in vivo study monitored different hematological parameters and examined blood film after oral administration of CTR-loaded niosomes and bilosomes compared with the traditional drug solution. The results of this in vivo study can indirectly reflect the oral bioavailability of CTR-encapsulated niosomes and bilosomes relative to that of CTR solution.

## 2. Results and Discuss

### 2.1. Chromatography

**Figure 1:**
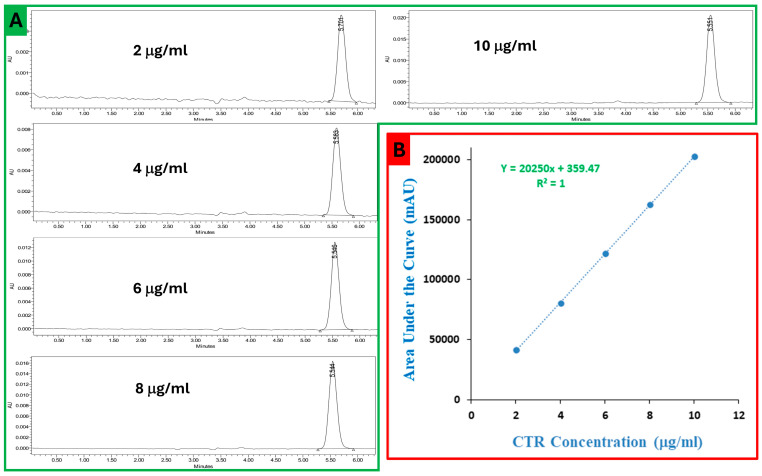
(**A**) Chromatograms of calibration standards of CTR in different concentrations (2, 4, 6, 8 and 10 μg/mL); and (**B**) the constructed calibration curve between area under the curve (AUC) of the peak (mAU) and CTR concentration (μg/mL).

Figure 1A shows representative chromatograms obtained at 270 nm for cytarabine working standards prepared in different concentrations (2, 4, 6, 8, and 10 μg/mL). From the chromatograms, the peak of CTR was eluted after 5.5 min, and these peaks show a symmetric appearance. The calibration curve was constructed and is presented in Figure 1B, with the regression equation and R^2^ value. It is clear from the calibration curve that there is a linear relationship from 2 to 10 μg/mL. This was further supported by the correlation coefficient value, which was 1 in the straight-line best-fitting equation. The obtained equation of linear regression was Y = 20,250x + 359.47, where Y is the measured area under the curve and x represents CTR concentration. Also, the percent recovery was recorded as 101.3–103.4% for interday accuracy and 99.1–101.3% for intraday accuracy, indicating acceptable accuracy according to ICH guidelines. The precision was acceptable, as the interday data’s relative standard deviation (RSD) was between 0.3 and 3.2% and 0.5 and 2.4% for intraday data. The limit of detection (LOD) was found to be 0.1 μg/mL, and the limit of quantification (LOQ) was 0.18 μg/mL.

### 2.2. Particle Size and Zeta Potential

The particle size values were expressed as Z-average and presented in Table 1. The average particle size of CTR-niosomes was 152 nm and was 204.3 for CTR-bilosomes. This data revealed that CTR-bilosomes were significantly (*p* < 0.01) larger than CTR-niosomes, reflecting the contribution of charged surfactant to increased vesicle size. Other investigators reported similar results of increased particle size of bilosomes using variable ratios and methods of preparation [25,26]. The particle size distribution index (PDI) reflected heterogeneous distribution for both formulations, which is expected with the current preparation technique that involves vesicle size reduction by bath sonication (Table 1). Zeta potential values (Table 1) were found to be −42.8 for CTR-niosomes and −87.8 mV for CTR-bilosomes, which is predicted due to the negative charge nature of bile salts in contrast with the non-ionic nature of tween 80, but both formulations have acceptable dispersibility. A negative charge on the surface of non-ionic surfactants containing vesicles was widely reported [27,28]. This negativity originated from the documented tendency of lipids to adsorb the aqueous hydroxyl (OH^−^) ion by hydrogen bonding, which can polarize in aqueous dispersion carrying a delta-ve charge [29,30]. It is important to note that future vesicle size and zeta potential determination are recommended for bar vesicles to study the effect of drug encapsulation on these parameters.

### 2.3. Transmission Electron Microscopy

**Figure 2:**
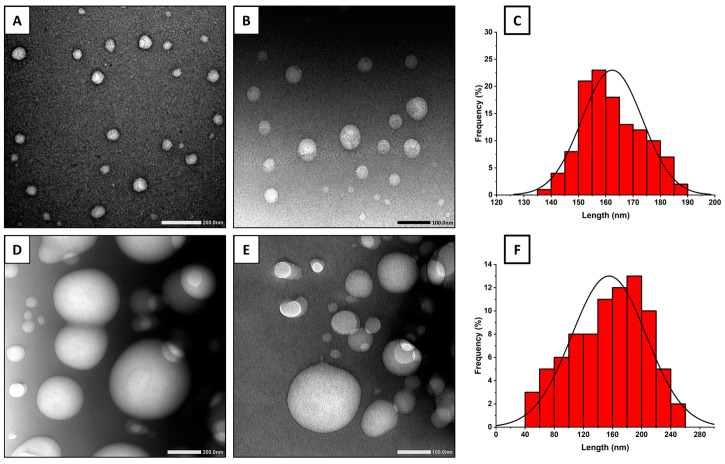
(**A**,**B**) Transmission electron micrographs of CTR-niosomes; (**C**) histogram representing size distribution of CTR-niosome in diameter frequency (%); (**D**,**E**) CTR-bilosomes; and (**F**) histogram representing size distribution of CTR-bilosome in diameter frequency (%).

Transmission electron micrographs of CTR-loaded niosomes and bilosomes are presented in Figure 2. CTR-loaded niosomes appeared as spherical vesicles with a size ranging from 70 to 100 nm. The CTR-loaded bilosome micrographs showed a similar morphology to niosomes, with vesicle sizes ranging from 110 to 200 nm. The trend of particle size distribution is similar to that recorded by photon correlation spectroscopy. Similar morphology and particle size were shown for niosomes and other vesicular carriers [31,32]. The discrepancy between the recorded size values of vesicles by photon correlation spectroscopy and TEM is expected. This is because sample preparation for TEM measurement involves dehydration and staining, which can affect the size. Moreover, it provides an average size for particles moving in dispersion, but TEM captures a specific field. A similar discrepancy was noticed in previous work and was similarly discussed [31].

### 2.4. Entrapment Efficiency Determination

In this parameter, we calculated how much the drug was entrapped in the formulations. Table 1 presents the value of entrapment efficiency of CTR in niosomes and bilosomes. The recorded values were 32.61% ± 4.4 for niosomes and 29.08% ± 4.8 for bilosomes. The recorded data correlate with the hydrophilic nature of CTR, which is believed to be localized in the aqueous core of the vesicles [33]. Thus, the limited entrapment efficiency of both vesicular systems can be related to the limited entrapped volume of the aqueous phase. A similar trend was obtained for the entrapment of hydrophilic drugs in liposomes with deformable sodium-cholate-containing vesicles showing relatively lower entrapment efficiency than non-deformable vesicles [34]. It is essential to highlight that the large particle size of bilosomes was not reflected as increased entrapment efficiency, which can be attributed to the fluidizing effect of bile salts on the bilayer structure of the vesicular membrane, which leads to loss of entrapped CTR [35]. Moreover, free bile salts in a dispersion medium can induce micellar solubilization of the drug outside the vesicles, further reducing CTR entrapment [36]. Drug loading (DL%) values were computed, and the results indicated that CTR-niosomes and CTR-bilosomes were able to load amounts of CTR equal to 0.156 and 0.134% of lipid components, respectively.

### 2.5. In Vitro Release of CTR

**Figure 3:**
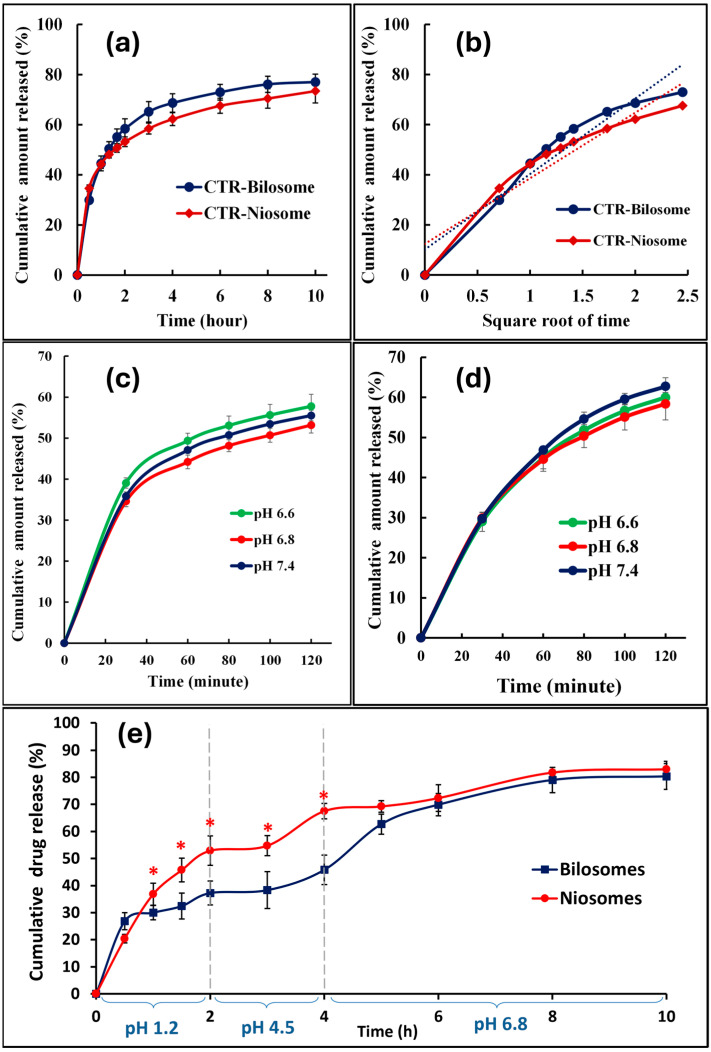
Release profiles of cytarabine from (**a**) CTR-niosomes and CTR-bilosomes at pH 6.8 for 10 h; (**b**) Fitting of release profiles of the tested niosomes and bilosomes at pH 6.8 with Higuchi release kinetic model; (**c**) the CTR-niosomes at different intestinal pH, including 6.6, 6.8, and 7.4 for 2 h; (**d**) CTR-bilosomes at different intestinal pH, including 6.6, 6.8, and 7.4 for 2 h; and (**e**) CTR-niosomes and CTR-bilosomes at continuous pH variation medium for 10 h. * Amounts of CTR release from niosomes are significantly higher than that recorded from bilosomes at corresponding time points.

The cumulative percent of CTR released was plotted against time, as presented in Figure 3. The kinetics of CTR release data were computed by fitting to the zero order, first order, and Higuchi models. Table 2 presents the correlation coefficient (R^2^) adopted to select the best fitting and the release kinetic model. The release rate from CTR-loaded niosomes and CTR-loaded bilosomes was comparable, with no significant difference in drug release rate. This is clear from the recorded values of the cumulative percent released (Figure 3a). The similarity in release was further confirmed after computing the F2 values using the similarity factor test (F2 = 65), which indicated no significant difference between the tested vesicular systems. Release data fitting indicated that the Higuchi model is the best kinetic model describing CTR release from the tested vesicular systems (Table 2). Ideally, Higuchi release kinetics are expected for matrix-based systems. The existence of Higuchi release in fluid systems like niosomes and bilosomes can be explained by considering the lamellar structure of these vesicles. Higuchi’s release supports the development of multilamellar vesicles with CTR entrapped in the aqueous compartments. This assembly allows drug liberation from surface lamellae, followed by diffusion from the internal to external lamella. This will create a concentration gradient, and drug release will depend on the diffusion through successive lamellae. Similar findings and explanations were recorded for the vesicular system [31].

This investigation was extended to better understand CTR liberation from tested formulations throughout different segments of GIT. Accordingly, release behavior was monitored at continuous pH variation, mimicking the in vivo pH status. The recorded release profile is shown in Figure 3e. CTR release from niosomes showed continuous pH-independent release. Contrarily, CTR release from bilosomes depended on pH with an initial rapid release, which tailed off with time in the acidic environment (pH 1.2 and 4.5) to record significantly lower release compared with niosomes at the corresponding time points (*p* < 0.05), as shown in Figure 3e. The difference in release pattern was gradually abolished after adjusting the pH to 6.8 to provide comparable cumulative amounts of release eventually. This pattern of controlled drug release of bilosomes in an acidic environment was previously reported [37]. Other investigators using various drugs recorded the pH-responsive release behavior of bilosomes [25,38].

The effect of pH variation on CTR release from niosomes and bilosomes was investigated, and the recorded release profiles are shown in Figure 3c,d. This employed different pH values, which represent the environment in tested intestinal segments, including jejuno-ileum (pH 7.4), duodenum (pH 6.6), and colon pH (6.8). The recorded release data revealed similar release rates from either niosomes or bilosomes at different intestinal pH, as indicated by the similarity factor test (F2 value ranged from 72–90). These results suggested that the pH value did not significantly affect the release rate of the drug from the tested vesicular system. In addition, the release kinetics revealed in Table 2 indicated the best fit for the Higuchi model at all the tested pH values.

### 2.6. Single-Pass In Situ Intestinal Perfusion Study

**Figure 4:**
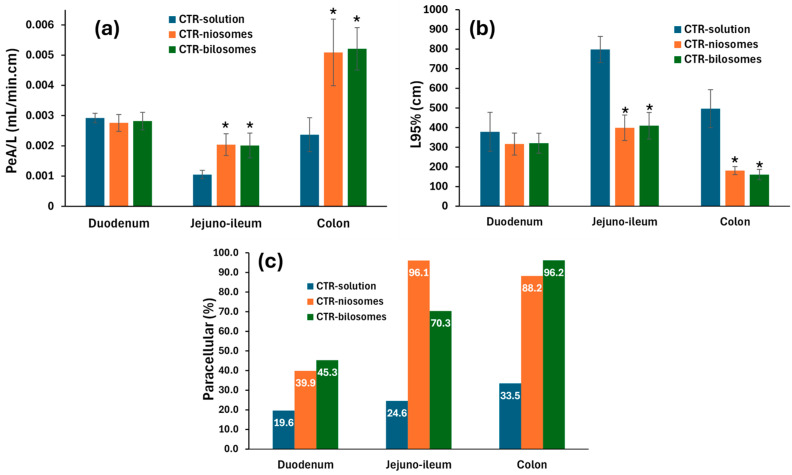
Bar charts of (**a**) absorptive clearance; (**b**) L95%; and (**c**) percent of paracellular pathway in intestinal absorption of CTR passing different segments of GIT in the form of CTR solution (blue), niosomes (CTR-niosomes) (orange), and bilosomes (CTR-bilosomes) (green). Values are expressed in mean ± SD (n = 4). * Significantly different compared to the corresponding CTR solution. *p* < 0.05 is considered significant.

Intestinal absorption of CTR was monitored to assess the dependence of its absorption on the absorption site and to predict the pathways and mechanisms of absorption. Furthermore, the effects of niosomal and bilosomal encapsulation on intestinal absorption were tested. The rabbit in situ intestinal perfusion technique was selected for this study based on previous surveys that indicated the practicability of this model in assessing intestinal absorption [39,40,41]. The computed permeation parameters are presented in Table 3 and Figure 4. The permeation data indicate incomplete absorption from the small intestine. This is clear from the recorded L95% values, which were 378.45 cm, 797.67 cm, and 496.29 cm for the duodenum, jejuno-ileum, and colon, respectively (Figure 4b). This was further shown by the recorded negative values for the ARL in all tested segments. Other permeation parameters delivered the same message (Table 3). The permeation data also highlighted site-dependent absorption from the small intestine, with the absorption rate decreasing distally. This is evident from the PeA/L values, which were 0.00292 and 0.00105 mL/min.cm in the duodenum and jejuno-ileum (Figure 4a). The absorptive clearance per unit length was increased in the colon compared with the jejuno-ileal segment (Figure 4a). This pattern suggests the existence of a possible role for intestinal efflux transporters. This suggestion is based on the site-dependent expression of P-gp, abundant in the distal parts of the small intestine [42]. The recorded data comply with literature reports that classified CTR as a substrate for intestinal efflux [5]. The polar nature of CTR can add to the poor membrane permeability [6]. Also, the poor permeability of CTR magnified the influence of P-gp efflux transporters. Multiple studies demonstrated that the significance of the efflux action of P-gb is inversely correlated with the drug’s tendency to passive diffusion toward intestinal cells [43]. These factors explain the poor absorption behavior of CTR after oral administration. Notably, the absorption pathway of CTR depended on the absorption site in such a way that CTR is mainly permeated via the transcellular route in all segments, with the contribution of the paracellular pathway increasing distally (Figure 4c). This pattern is expected considering the increase in water flux from the distal part of the small intestine with maximum water absorption in the colon and the fact that the tight junction showed minor expression in colon epithelia [44].

Encapsulation of CTR in niosomes or bilosomes showed site-dependent enhancement in intestinal absorption of CTR. This enhancement was reflected only as a trend in the duodenal segment, as indicated from the recorded L95% values after perfusion of vesicular systems compared with a simple CTR solution. The enhancement became statistically significant (*p* < 0.05) in the jejuno-ileum and colon, as indicated by the significant increase in the absorptive clearance per unit length and the fraction absorbed of CTR from niosomes and bilosomes compared with the drug solution (Figure 4a). This was also reflected as a significant reduction in L95% (*p* < 0.05) computed after perfusion of vesicular formulations compared with the corresponding simple aqueous solution (Figure 4b). Surprisingly, the relative contribution of the paracellular pathway in intestinal absorption of CTR was increased after delivery from niosomes and bilosomes. This was clearer in the case of the jejuno-ileum and colon (Figure 4c). Such behavior may suggest possible vesicular invasion through the tight junction, which requires further investigation. Also, translymphatic delivery of vesicular systems has been suggested [31]. A link between water flux and lymph flow was reported [45]. The relationship between increased water flux and increased paracellular contribution in oral delivery was noted, hypothesizing that the enhanced bioavailability of CTR-niosomes and CTR-bilosomes could be attributed to increased water flux (Table 3) [46]. However, the increased water flux was also reported to enhance mesenteric lymph transport by the solvent drag effect, leading to increased drug appearance in lymphs [47]. These two suggestions can be compatible, considering that increased intestinal water flux can stimulate mesenteric lymph flow, dragging fluid from the intestinal lumen through intercellular gaps of the intestinal lining. Similar discussions were reported previously using different experimental models [45,48]. This can explain the apparent increase in the relative contribution of paracellular transport of CTR after niosomal and bilosomal encapsulation. Vesicular systems like liposomes showed high potential for enhanced intestinal absorption [5]. The mechanism of enhanced intestinal absorption after vesicular delivery was postulated, and alternative possibilities were presented. The first depended on intact vesicular delivery via the lymphatic system, evading the hepatic metabolism [35]. This postulation is applicable here considering the increased correlation between permeation and water flux after vesicular delivery, apparently expressed as an increase in the fraction absorbed via the paracellular pathway. Translymphatic transport can probably avoid the P-gp efflux, which is also advantageous in the case of CTR. Another explanation for enhanced intestinal absorption from niosomes and bilosomes is the fluidizing effect of vesicular components. This can affect both trans and paracellular pathways, as it might also loosen the cellular junction [49]. This fluidizing effect can explain enhanced drug absorption even after being released from the vesicles. The postulated mechanisms can thus cover the absorption of the entrapped CTR and the fraction released. This reflects the harmony between the in vitro release data and the in situ intestinal absorption.

### 2.7. In Vivo Evaluation of Anti-Leukemic Effect

#### 2.7.1. Hematological Analysis

**Figure 5:**
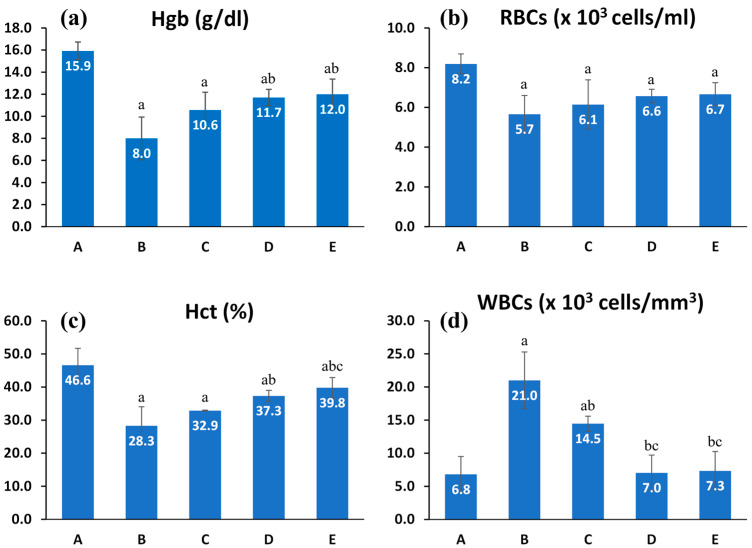
Comparative effect of CTR solution (group C), CTR-niosomes (group D), and CTR-bilosomes (group E) on (**a**) hemoglobin (Hgb); (**b**) red blood cell counts (RBCs); (**c**) hematocrit (Hct); and (**d**) white blood cell counts (WBCs). All graphs are compared to the healthy group (group A) and the diseased untreated group (group B). Values are expressed in mean ± SD (n = 5). *p* < 0.05 is considered significant. a: significant compared to group A. b: significant compared to group B. c: significant compared to group C.

This experiment was conducted to evaluate the effect of niosomal and bilosomal encapsulation on the therapeutic effect of CTR on leukemia. This was taken as an indirect measure for enhanced intestinal absorption and, hence, the bioavailability after vesicular delivery. Hematological parameters for all tested groups are presented in Figure 5 and Table 4. Group B (+ve control group) showed marked changes compared to group A (healthy group) as observed in WBC count, which was found to be 21.00 × 10^3^ cells/mm^3^. This three-fold increase was significantly (*p* < 0.05) higher than that of the healthy group (6.80 × 10^3^ cells/mm^3^) (Figure 5d), and results also reflected about two-fold reduction of hemoglobin, RBC count, and hematocrit values, which were 8.00 g/dL, 5.66 × 10^6^ cells/μL, and 28.27%, respectively, which were significantly (*p* < 0.05) lower than corresponding parameters in the healthy group (Figure 5a–c). These significant changes in blood parameters indicated successful leukemia induction by benzene in the tested groups. Other investigators reported similar results for benzene-induced leukemia [50,51]. The use of hematological studies to evaluate acute myeloid leukemia was established earlier [52]. This originated from the fact that all types of blood cells are affected by bone marrow activity [53]. Moreover, this type of analysis can be used to screen prognostic parameters in humans [54,55].

Groups C, D, and E were treated with CTR solution, CTR-niosomes, and CTR-bilosomes, respectively. Hemoglobin level was improved by 32% after treatment with CTR solution, reaching 10.57 g/dL for group C, but this improvement was insignificant (*p* > 0.05). Meanwhile, groups D and E, treated by CTR-niosomes and CTR-bilosomes, showed significant improvement in hemoglobin level (*p* < 0.05) with 46% and 50% improvement, respectively, compared to the untreated diseased group. However, it should be noted that complete recovery to the normal hemoglobin level was not attained (Figure 5a). The RBCs showed only a trend of increased count after treatment compared with the untreated diseased animals, and the vesicular systems were ranked slightly better than the simple CTR solution (Figure 5a).

Hematocrit is a parameter representing the percentage of cellular composition in blood volume. The results indicated about 16% improvement after CTR solution treatment, insignificant (*p* > 0.05) compared with the untreated diseased group. Treatment with niosomes or bilosomes resulted in significant improvement (*p* < 0.05) in the hematocrit level, with 32% and 41% improvement, respectively, compared to the untreated group. Concerning the rank of vesicular systems, bilosomes were ranked better than niosomes (Figure 3c).

Concerning white blood cell count treatment with CTR solution (group C), the WBCs were significantly reduced by 31%, reaching 14.45 × 10^3^ cells/mm^3^, compared to the untreated diseased group (group B). Despite significantly reducing WBC count after treatment with CTR solution, the WBCs remained significantly higher (*p* < 0.05) than the normal values. This reflects poor oral bioavailability of CTR from simple aqueous solution, which agrees with the above-mentioned poor permeability in different intestinal segments. Treatment with niosomes or bilosomes (group D and E, respectively) resulted in 66 and 65% reductions in the WBC count to reach 7.34 and 7.025 × 10^3^ cells/mm^3^ for CTR-niosomes and CTR-bilosomes, respectively, compared to the untreated diseased group, and this reduction was found to be significant (*p* < 0.05). Furthermore, these values were comparable to the normal group and were significantly lower than those recorded after treatment with CTR solution. It is important to note that the white blood cell count obtained by autoanalyzer can be affected by blast cell count, which is expected to be found in AML samples. Thus, increased WBCs can be attributed mainly to the increase of blast cells in the specimen [56,57,58]. The improvement in the previous hematological parameters was also reflected in MCV and MCH values (Table 4).

The recorded data indicated the positive effects of niosomes and bilosomes for enhanced oral delivery of CTR to treat acute myeloid leukemia. This can provide evidence about the improved intestinal absorption process bypassing variable limiting barriers (poor permeability, intestinal metabolism, and P-gb) and is correlated with our intestinal perfusion study data.

#### 2.7.2. Blood Film Examination

**Figure 6:**
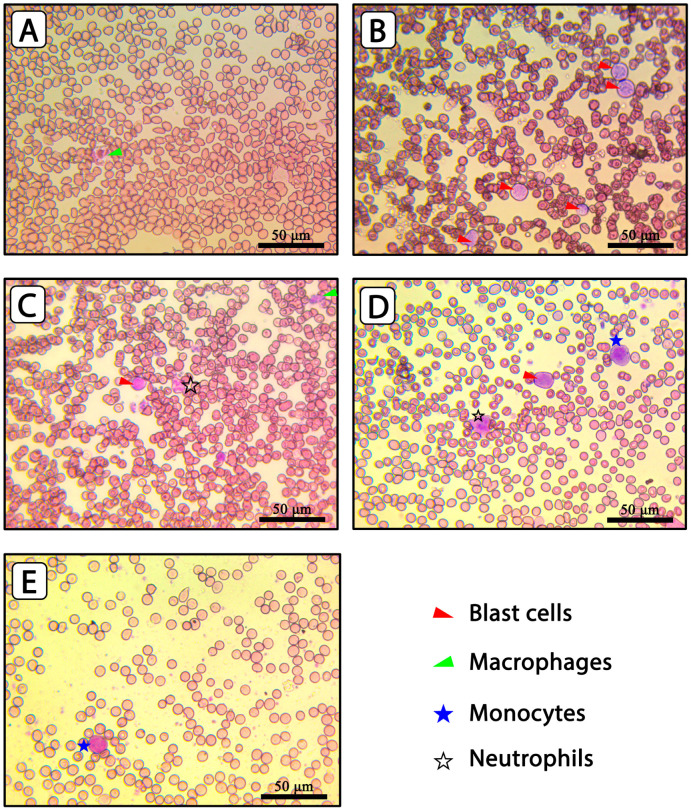
Blood film pictures represent animals in (**A**) the normal group; (**B**) diseased group; (**C**) the group treated with CTR solution; (**D**) CTR-niosomes; and (**E**) CTR-bilosomes.

Figure 6 illustrates micrographs of blood films for all tested groups. The examinations were completely compatible with results obtained from the hematological analysis of the normal group (A). Blood films of rats from the diseased group (group B) showed numerous blast cells in the field. As presented in Figure 6B, blast cells were predominant in the field, which appeared as cells with a high nucleus-to-cytoplasm ratio (large nucleus) and dotted appearance, representing immature chromatin with the absence of cytoplasmic granules. Blast cells should not be observed normally in the peripheral blood film, and their presence in group B blood films indicated the successful induction of leukemia [59]. The blood films prepared from treated groups showed decreased blast cell availability. Group C (Figure 6C), representative of rats treated with CTR solution, showed fewer blast cells than the diseased untreated group. Further reduction in blast cells was shown after niosomal or bilosomal therapy, with the latter showing marked improvement (Figure 6D,E). These results, along with the hematological studies, confirmed the tested vesicular systems’ ability to enhance CTR’s therapeutic efficiency. This can be due to improved intestinal absorption and bioavailability after oral administration. Finally, it is essential to emphasize that another control was required for such a study in which drug-free niosomes and bilosomes are administered to test the effect of formulation components. Previous publications indicated the absence of anticancer activity for niosomes, supporting our experimental design [60,61]. However, we recommend including this control in future investigations.

## 3. Materials and Methods

### 3.1. Materials

CTR powder was donated as a research sample from Hikma Specialized Pharmaceuticals, Cairo, Egypt. Sorbitan stearate (Span 60) and bile salts were acquired from Sigma Chemical Company, St. Louis, MO, USA. Tween 80 and isopropyl alcohol were purchased from bioChem Chemicals Company, Cairo, Egypt. Cholesterol, ethanol, monobasic, and dibasic phosphate buffer components were procured from El-Nasr Pharmaceutical Chemicals Company, Cairo, Egypt.

### 3.2. Chromatography

The experimental samples were analyzed quantitively by high-pressure liquid chromatography (HPLC) employing the Waters system (Waters Alliance 2695, Milford, MA, USA). This HPLC system was supported by a Photodiode array detector (996 PDA) and an auto-sampler. The mobile phase involved online mixing of filtered phosphate buffer solution (pH 7) and methanol at a ratio of 9:1. A sample of 10 μL was injected with the mobile phase through ODS-3 reversed-phase column (Inertsil^®^ 25 cm × 0.46 cm, GL Sciences Inc., Tsukuba, Japan). This column has an average particle size of 5 μm. PDA detected the effluent at 270 nm, and the drug concentration was determined. This method was completely validated to determine the accepted range, linearity, accuracy, precision, and limit of detection and quantification.

### 3.3. Preparation of Niosomes and Bilosomes

This employed the already established method for preparing niosomes [31]. Firstly, CTR powder was dissolved in ethanol and sonicated for 30 min to obtain a clear solution (2.5 mg/mL). For noisome and bilosome preparation, the formulations’ components described in Table 5 were heated together in a water bath at a temperature of 65 °C ± 1. Then, the drug ethanolic solution was added and stirred gently until clear dispersion was obtained. After that, an equal amount of preheated water (4 mL) was added with continuous stirring on the water bath till clarity. Once the dispersion was obtained, the volume was completed gradually with water at the same temperature to a final volume (50 mL), producing niosome and bilosome dispersions containing CTR at 200 µg/mL concentration. This dispersion was kept at room temperature overnight to confirm complete hydration. The next day, the dispersion was sonicated for 30 min using a bath sonicator (Elmasonic S 60, Elma Schmidbauer GmbH, Gottlieb-Daimler-Str, Singen 78224, Germany). This hydrated dispersion contains the vesicular system, which is believed to keep its vesicular structure as far as it remains in aqueous dispersion.

### 3.4. Photon Correlation Spectroscopy

After proper dilution with filtered water, all formulations were analyzed for particle size and zeta potential using a dynamic light scattering technique. Tested samples were loaded into a specially designed cuvette and tested by Zetasizer Nano series (Nano-ZS, Malvern Instruments, Malvern WR14 1XZ, UK). All samples were tested at ambient temperature in triplicate.

### 3.5. Transmission Electron Microscopy

The size and morphology of the prepared nano-systems were examined using transmission electron microscopy. The prepared dispersions were diluted with a suitable amount of filtered water and sonicated for 10 min. Then, each dispersion was stained with uranyl acetate to increase the contrast of the micrographs before scanning by transmission electron microscope JEM-1400 Plus, JEOL USA Inc., Peabody, MA, USA.

### 3.6. Entrapment Efficiency and Drug Loading Determination

The entrapment efficiency (EE%) was calculated after the complete separation of the amount of free CTR by the dialysis sacs method [62]. Briefly, dialysis sacs were prepared by cutting cellulose tubing (molecular weight cut-off = 12,000 Daltons with an average flat width of 25 mm, Sigma-Aldrich, St. Louis, MO 63103, USA) into sacs each of 7 cm length. These sacs were soaked in distilled water for 24 h to ensure complete swelling. The sacs were sealed precisely from one end by a cotton thread, and then 2 mL of niosomes or bilosomes (3 replicates of each) were loaded into the sac and similarly sealed from the other end. The dialysis medium was water (100 mL), where the sacs were immersed for 4 h at room temperature to ensure that all free CTR was separated. The dialysate was then collected and quantified for CTR concentration using the previously described HPLC method. The total amount of free CTR in the dialysate was employed for entrapment efficiency calculation using the following equation.

$$EE\;(\%)=\frac{Total\;amount\;of\;CTR\;in\;2\;mL\;of\;formulation-amount\;of\;free\;CTR}{Total\;amount\;of\;CTR\;in\;2\;mL\;of\;formulation}\times 100$$

Drug loading (DL) represents how much CTR can be loaded per lipid weight. This was calculated by dividing the amount of CTR in each formulation, calculated in mg, by the total weight of lipids added, which was calculated in grams.

To calculate the drug loading value of CTR-niosomes and CTR-bilosomes, the following equation was used [63,64]:

$$Drug\;loading\left(DL\%\right)=\frac{Entrapped\;CTR(g)}{weight\;of\;S60+Ch+T80/BS(g)}\times 100$$

where S60: Span 60, Ch: cholesterol, T80: Tween 80, and BS: Bile Salts.

### 3.7. In Vitro Drug Release Study

The release profile of CTR from niosomes and bilosomes was determined using a dialysis sac immersed in release media with continuous shaking at a biological temperature [24]. Briefly, 3 mL of niosomes/bilosomes were placed in the previously tied dialysis sac of M.W cut off 12,000 D (Cellulose tubing, Sigma Diagnostics, St. Louis, MO 63103, USA). In a beaker, 50 mL of phosphate-buffered saline pH 6.8 was filled and conditioned for 15 min at 37 °C in a thermostatic shaking incubator (Small Capacity Thermostatic Shaking Incubator (BJPX-200N), Biobase Co., Jinan 250101, China). The dialysis sacs were tied from their end and immersed in beakers inside the incubator, and the beakers were occluded with aluminum foil. The beakers were subjected to continuous shaking at 80 rpm speed at 37 °C. Samples were analyzed at predetermined intervals (30 min, 60 min, 80 min, 100 min, 2 h, 3 h, 4 h, 6 h, 8 h, and 10 h), and fresh medium was replenished to ensure sink condition. The samples were analyzed by HPLC to determine the amount of CTR released at every time point, and the percent released was plotted as a function of time intervals.

As CTR-niosomes and CTR-bilosomes are developed for oral administration, the release behavior was monitored using continuous pH variation to cover pH values of 1.2, 4.5, and 6.8. The experiment initially started using 0.1 N HCl as a release medium (pH 1.2) for the first two hours. Then, the pH was elevated to pH 4.5 for the next two hours by adding phosphate buffer salts (189 mg KH_2_PO_4_ and 247 mg Na_2_HPO_4_ for each 50 mL of release medium) and adequate drops of 1 N NaOH. After that, the pH was changed to 6.8 by 1 N NaOH for the rest of the experiment. Samples (2 mL) were collected after 0.5, 1, 1.5, 2, 3, 4, 5, 6, 8, and 10 h [65].

The release study was also extended to monitor the effect of pH variation on CTR release as a correlation parameter with the in situ intestinal environment where the pH is varied in each region (jejuno-ileum pH 7.4, duodenum pH 6.6, and colon pH 6.8). This study adopted the previously mentioned release conditions, but the release medium was phosphate-buffered saline adjusted to variable pH values (6.6, 6.8, and 7.4), and samples were collected at 30, 60, 80, 100, and 120-min intervals.

### 3.8. Single-Pass In Situ Intestinal Perfusion Study

#### 3.8.1. Perfusion Fluid Composition

A simple aqueous solution of CTR was employed as a control. This was prepared by dissolving a known amount of CTR in phosphate-buffered saline (PBS) before suitable dilution with PBS to develop a 25 μg/mL concentration. The pH of the perfusion fluids was adjusted at 7.4 for the jejuno-ileum, 6.6 for the duodenum, and 6.8 for the colon. Perfusion fluids containing CTR-loaded niosomes and bilosomes were prepared by dilution (1 in 8) of the vesicular formulations by PBS adjusted to the required pH value, followed by bath sonication for 30 min. This produces niosomal dispersion containing 25 μg/mL of CTR. In all cases, the prepared perfusion fluid was temperature controlled to 37 °C throughout the experiment.

#### 3.8.2. Intestinal Perfusion Study

The ethical committee, Faculty of Pharmacy, Tanta University, approved this work’s animal procedures and protocol by approval number (TP/RE5/23 Ph-6) and approval date 26 May 2023. The experiment used eighteen male albino rabbits (2–2.5 kg). The rabbit was used as a model animal for in situ intestinal perfusion studies based on previous studies indicating this strategy’s feasibility for monitoring intestinal absorption. In addition, the use of rabbits ensures the existence of anatomical and physiological characteristics similar to those of the human intestine. It permits the investigation of site-dependent absorption from different intestinal segments. It allows the calculation of the permeability surface area product and other permeation parameters. These calculations are applicable for comparative studies between various formulations, as in the current study [66,67]. Initially, the fasting rabbit was anesthetized by an intramuscular injection of ketamine HCl (45 mg/kg), and an extra dose (25 mg/kg) was given if needed. Xylazine HCl was injected intramuscularly (10 mg/kg) 15 min before ketamine injection to avoid any possible convulsion during anesthesia.

The surgical procedures were performed after fixing the rabbit in the supine position on a thermostatted mattress. The target area of the abdomen was shaved, and a midline longitudinal abdominal incision was made. The targeted segment was exposed, the required length was measured, and the segment was ligated by surgical suture to be ready for canulation. Both segment sides were canulated and perfused with the previously conditioned PBS for cleaning.

Once the tested segment was ready, the perfusion solution was pumped using a constant-rate perfusion pump (Harvard-22, Harvard Apparatus, Millis, MA, USA) at 0.27 mL/min. Samples eluted from the segment under investigation were collected every 10 min for 2 h. The volume of each collected sample was determined and then centrifuged to develop a clear solution suitable for HPLC analysis to determine CTR concentration. Niosome and bilosome perfusates were dispersed vesicles that required proper dilution with isopropyl alcohol to solubilize vesicle components, forming a clear solution before the HPLC assay.

#### 3.8.3. Data Analysis and Interpretation

The raw data obtained from all perfusion procedure intervals were used to calculate different permeation parameters of CTR together with the water flux. The net water flux is the difference between the expected volume (flow rate × time) and the actual volume of perfusate measured at the end of each interval. Calculations of permeation parameters allow prediction of the mechanism of drug passage through the intestinal wall.

##### Absorptive Clearance

The concentration of the eluted perfusate was used to determine how much CTR permeates the intestine, but this should be corrected firstly by water flux to neutralize the apparent high concentration resulting from water flux. The ratio between these corrected data of perfusate concentration (C_out_) and the starting concentration of CTR that entered the segment (C_in_) was used to measure the remaining fraction of CTR in the perfusate at each time point. This was used to calculate the steady-state fraction remaining {C_out_/C_in_}_ss_ by taking the mean of the remaining fraction at every time point in the second hour of the experiment. From this parameter, along with the average flow rate (Q, mL/min) in these time points, we can use Equation (1) to determine the permeability surface area product (Pe.A, mL/min), which is known as absorptive clearance and is representative of the permeability coefficient of CTR (Pe, cm/min) multiplied by surface area (A, cm^2^).

(1) $${\left\{C_{out}/C_{in}\right\}}_{SS}=e^{-(Pe.A/Q)}$$

This equation can be simplified by rearrangement into Equation (2), which is used in our calculations.

(2) $$Pe.A=-Q\times \ln{\left\{C_{out}/C_{in}\right\}}_{SS}$$

Accordingly, we can use these parameters not only for the deduction of the fraction remaining; we can also utilize these data to compute the fraction absorbed of CTR (Fa) in the tested segment from Equation (3).

(3) $$Fa=1-{\left\{C_{out}/C_{in}\right\}}_{SS}$$

From these data, we can also calculate the apparent length of the segment required for complete absorption of CTR (l*, cm). This parameter was then used to detect anatomical reserve length (ARL), which clarifies how much of the actual anatomical length of the segment (L*) is required for complete CTR absorption. The problem was that complete absorption could not be reached because of the logarithmic nature of the absorption process. So, we assumed that complete absorption could be approximately attained when 95% of CTR was absorbed and only a minute amount (5%) of the drug was still in the intestinal lumen. The length required for 95% absorption (L95%) can be easily calculated from Equation (4), which is like the absorptive clearance equation, and then we can calculate ARL from Equation (5) [24], where (l*) represents L95% of CTR in the intestinal segment.

(4) $$0.05=e^{-(Pe.A\times l*/Q)}$$

(5) ARL=L*−l*

##### Effect of Water Flux on CTR Intestinal Absorption

The effect of water flux is the determinant parameter to predict the mechanism of CTR absorption. The paracellular absorption pathway is linked to water flux. This was taken as the base for predicting the absorption pathway of CTR. The first step of data analysis was to calculate the net water flux (J_w_) from the difference between the volume of fluid that entered the segment during the time interval (Q_in_) and the volume of perfusate collected from the end of the segment during the same time interval (Q_out_).

(6) $$J_{w}=Q_{in}-Q_{out}$$

This parameter was then normalized to unit segment length (J_w_/l). The relative contributions of paracellular and transcellular pathways in intestinal absorption were then calculated. This involved plotting absorptive clearance per unit length (Pe.A/l) as a function of J_w_/l, followed by linear regression. The extrapolation of the regression line will meet with the Y axis at a point that represents the absorptive clearance at zero water flux. This accounts for transcellular absorption. Accordingly, dividing the intercept by the average Pe.A/l and expressing it as a percentage will provide the % transcellular. The percentage paracellular can be calculated by difference [67].

### 3.9. In Vivo Evaluation of Anti-Leukemic Effect

#### 3.9.1. Ethics Statement

This research was performed after approval by the ethical committee of the faculty of pharmacy, Tanta University on experimental design and animal treatment with approval number (TP/RE5/23 Ph-6).

#### 3.9.2. Experimental Animals

This in vivo study utilized twenty-eight male albino rats weighing 110 to 130 g. All rats were acclimatized firstly in the normal environment, with a regular, freely accessible diet and 12 h of dark/light cycles, with housing in standard plastic cages for 2 weeks.

#### 3.9.3. Experimental Groups

Rats were divided into five groups: group A (n = 4) is a negative control group where rats are neither induced for leukemia by benzene nor treated with any form of CTR. Rats in this group received only corn oil via subcutaneous injection during the induction period. Group B (n = 6) was a positive control group that was subcutaneously injected with benzene during the induction period, but it did not receive any treatment. Group C (n = 6) contained rats subjected to leukemia induction by benzene during the induction period and treated with CTR solution during the treatment period. Group D (n = 6) was the group of rats that underwent leukemia induction by benzene and were treated with CTR-loaded niosomes. Finally, rats in group E (n = 6) were subjected to leukemia induction by benzene and treated with CTR-loaded bilosomes.

#### 3.9.4. Leukemia Induction

**Figure 7:**
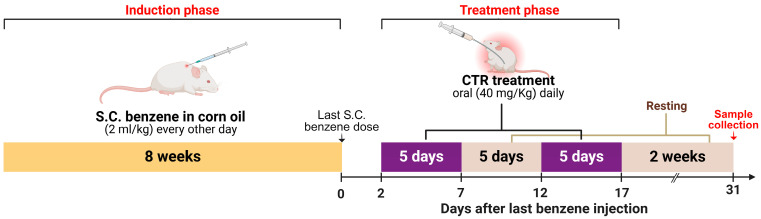
Schematic illustration of experimental design of in vivo assessment of the anti-leukemic effect of CTR solution, CTR-niosomes, and CTR-bilosomes.

Acute myeloid leukemia (AML) was induced utilizing the benzene-induced leukemia model. This was achieved by subcutaneous injection of benzene (2 mL/kg) diluted in corn oil (1:1 *v*/*v* dilution), which was injected every other day for 8 weeks [68,69]. Once the induction was obtained (based on the blood picture), the treatment protocol was started two days after the last subcutaneous benzene dose (Figure 7).

#### 3.9.5. Treatment Protocol

Treatment by CTR was received in cycles; i.e., five days of treatment and five days resting. This experiment included two cycles over the treatment period. The drug solution, niosomes, and bilosomes were prepared in the same concentration, and each rat received the drug orally with a dose of 40 mg/kg once daily during the planned days for administration (Figure 7) [70].

#### 3.9.6. Hematological Analysis and Blood Film Examination

Two weeks after the end of the second cycle, blood samples were collected from the retro-orbital sinus by a heparinized capillary tube. About 1.5 mL from each animal was drained in an EDTA tube. These samples were analyzed via auto-analyzer for hematological parameters (RBCs, hematocrit, hemoglobin, and platelet count) [71].

At the same time, a fresh drop was collected from the tube and spread over a microscopic slide, making a thin blood film freshly dehydrated by methanol, stained by Leishman stain for 20 min, and finally rinsed with phosphate-buffered saline (PBS). Blood film from each group was examined under the microscope for blast cells [68].

### 3.10. Statistical Data Analysis

Statistical analysis employed a one-way ANOVA test followed by Tukey’s multiple comparisons as a post hoc test to determine the significance between groups. This analysis was performed utilizing Statistical Package for Social Sciences (SPSS) (SPSS Inc., Chicago, IL, USA), software for Windows, version 20.

## 4. Conclusions

CTR-loaded niosomes and bilosomes were successfully formulated with optimum particle morphology, size, and loading efficiency for such a hydrophilic drug. The drug was liberated via diffusion-dependent release, reflecting the multilamellar structure of the prepared vesicles. The intestinal absorption of CTR solution was unfavorable from distal parts of the intestine. Niosomes and bilosomes significantly enhanced CTR absorption from the jejuno-ileum and colon. Benzene-induced leukemia was accomplished, and leukemia induction was evident from the modulated blood picture and blast cells in the blood film. Niosomes and bilosomes enhanced the anti-leukemic effect of CTR compared with the corresponding drug solution. Niosomes and bilosomes showed comparative efficacy in enhanced intestinal absorption, but the in vivo study showed better responses from bilosomes concerning improved blood parameters. However, this requires future verification. This study introduced niosomes and bilosomes as promising carriers for enhanced oral delivery of CTR. It is recommended that future in vitro cellular absorption studies be conducted to further clarify the mechanism of enhanced intestinal absorption from these vesicles. Also, the pharmacokinetics of the drug must be investigated after administration of these vesicles with reference to the drug solution. In addition, an extended in vivo study is recommended in which survival rates, body weight changes, or histopathological analyses are conducted.

## Figures and Tables

**Table 1:** The characteristics of the CTR-niosomes and CTR-bilosomes.

Material (g)	CTR-Niosomes	CTR-Bilosomes
Z-average (nm)	152.0 (3.8)	204.3 (5.8) *
PDI	0.424 (0.01)	0.538 (0.06)
Zeta potential (mV)	−42.8	−87.8
Entrapment Efficiency (%)	32.61 (4.4)	29.08 (4.8)
Drug Loading (DL%)	0.156 (0.021)	0.134 (0.022)

Values are expressed in mean ± SD (n = 3). * Significant difference *p* < 0.05.

**Table 2:** Correlation coefficient (R^2^) values obtained from linear regression analysis of cytarabine release data from niosomes and bilosomes fitted to different release kinetics models.

Formulation	Zero Order	1st Order	Higuchi
CTR-niosomes (at pH 6.8 for 10 h)	0.6041	0.7646	0.8516
CTR-bilosomes (at pH 6.8 for 10 h)	0.5958	0.6140	0.8482
CTR-niosomes (at pH 6.6 for 2 h)	0.7822	0.9027	0.9609
CTR-niosomes (at pH 6.8 for 2 h)	0.8048	0.9187	0.9710
CTR-niosomes (at pH 7.4 for 2 h)	0.8028	0.8882	0.9700
CTR-bilosomes (at pH 6.6 for 2 h)	0.9119	0.8976	0.9960
CTR-bilosomes (at pH 6.8 for 2 h)	0.8977	0.9056	0.9965
CTR-bilosomes (at pH 7.4 for 2 h)	0.9147	0.8899	0.9947

**Table 3:** Parameters of CTR membrane transport passing different segments of GIT in the form of CTR solution, niosomes (CTR-niosomes), and bilosomes (CTR-bilosomes).

		C_out_/C_in_	%Fa/L (% cm^−1^)	JW/L (×10^−3^ mL/min cm)	ARL (cm)
Duodenum	CTR-solution	0.850 (0.015)	15.03 (1.48)	0.00195 (0.00228)	−358.45 (99.17)
	CTR-niosomes	0.850 (0.013)	14.99 (1.31)	0.00246 (0.00004)	−296.28 (55.72)
	CTR-bilosomes	0.853 (0.014)	14.69 (1.41)	0.00195 (0.00099)	−300.82 (50.61)
Jejuno-ileum	CTR-solution	0.879 (0.015)	12.09 (1.45)	0.00085 (0.00069)	−617.67 (66.62)
	CTR-niosomes	0.779 (0.034)	22.11 * (3.37)	0.00183 (0.09318)	−219.16 (64.69)
	CTR-bilosomes	0.785 (0.037)	21.50 * (3.66)	0.00148 (0.00013)	−229.72 (67.54)
Colon	CTR-solution	0.915 (0.009)	8.49 (0.86)	0.00259 (0.00077)	−481.29 (96.84)
	CTR-niosomes	0.802 (0.037)	19.81 * (3.69)	0.00665 (0.00250)	−166.22 (20.74)
	CTR-bilosomes	0.791 (0.016)	20.89 * (1.62)	0.00647 (0.00105)	−145.98 (26.29)

Values are expressed in mean (SD) (n = 3). *p* < 0.05 is considered significant. * Significantly different compared to the corresponding CTR solution.

**Table 4:** Experimental animal groups and the recorded hematological parameters.

Group	Description	MCV fl/cell	MCH pg/cell
A	No induction + no treatment	56.80 (3.26)	19.48 (0.42)
B	Induction + no treatment	49.73 ^a^ (2.73)	14.03 ^a^ (1.18)
C	Induction + CTR solution treatment	59.20 ^b^ (2.40)	17.33 ^b^ (0.93)
D	Induction + CTR-niosomes treatment	56.90 ^b^ (3.21)	17.84 ^b^ (1.13)
E	Induction + CTR-bilosomes treatment	59.82 ^b^ (4.15)	18.02 ^b^ (1.26)

Values are presented as average with standard deviation between brackets. MCV: mean corpuscular volume. MCH: mean corpuscular hemoglobin. Values are expressed in mean ± SD (n = 5). *p* < 0.05 is considered significant. a: significant (*p* < 0.05) compared to group A. b: significant (*p* < 0.05) compared to group B.

**Table 5:** The composition of the tested niosomes and bilosomes.

Material (g)	CTR-Niosomes	CTR-Bilosomes
Span 60	1.2	1.2
Cholesterol	0.4	0.4
Tween 80	0.3	--
Bile salt	--	0.3
CTR-Ethanol *	4	4
Water to	50	50

* Ethanolic solution of cytarabine with a concentration of 2.5 mg/mL.

## Data Availability

The data presented in this study are available upon request from the corresponding authors. The data are not publicly available due to privacy restrictions.
